# Establishment of a method to measure the intracellular potassium ion concentration of brain tissue using a simple device

**DOI:** 10.3934/Neuroscience.2024018

**Published:** 2024-08-26

**Authors:** Takaya Iwamoto, Minori Fujita, Yukiko Futamata, Teruki Okada, Ryuta Morinaga, Airi Nishi, Toshihiko Kinjo, Koichi Kawada, Kyosuke Uno, Nobuyuki Kuramoto

**Affiliations:** Laboratory of Molecular Pharmacology, Faculty of Pharmaceutical Sciences, Setsunan University, Osaka, Japan

**Keywords:** potassium ion, ion meter, simple device, homeostasis of potassium ion, intracellular concentration of potassium ion, central nervous system tissue

## Abstract

Intracellular potassium ion (K^+^) concentration is higher than extracellular K^+^ concentration. Some cells maintain intracellular potassium levels by taking up extracellular potassium. However, investigating these details requires techniques to measure intracellular potassium concentrations. We established a method to easily measure intracellular potassium concentrations using a simpler electrode. The LAQUAtwin K-11 was capable of linearly quantifying potassium concentrations and was not affected by cellular constituents other than nucleic acids; however, it did not tolerate low temperatures. Interference caused by a high concentration of nucleic acids was eliminated by the addition of cations. It was also suggested that the concentration of nucleic acids in the cell suspension was not sufficiently high to interfere with the measurements. Intracellular potassium concentrations increased and decreased in response to extracellular potassium concentrations. Exposure to valinomycin did not decrease the potassium concentration, suggesting that re-uptake of the potassium released outside the cells occurred immediately. Additionally, potassium concentrations could be measured in the brain tissue homogenates using the device. This measurement method can track the relative changes in potassium concentration in cells under various conditions and in tissues of various disease models.

## 1. Introduction

Potassium ions (K^+^) are taken up by sodium pumps into every cell and are, therefore, maintained at a higher concentration inside the cell (~150 mM) than outside it (3.5–5.0 mM) [1],[2]. The intracellular concentrations slightly vary among cell types and consistently result from transport by numerous carriers. The K^+^ concentration gradient between the intracellular and extracellular spaces is important for membrane potential formation. The membrane potential of a cell approximately correlates with the equilibrium potential of K^+^ (Ek), which is defined by the K^+^ concentration inside and outside the cell. In addition, the opening of K^+^ channels on the cell membrane negatively regulates the cell membrane, causing hyperpolarization and suppressing the excitation of excitable cells such as muscle and nerve cells.

A significant increase or decrease of the K^+^ levels within an individual either induces hyperkalemia or hypokalemia, respectively, as observed in conditions such as renal failure or drug intake [1],[2]. Muscle abnormalities, including skeletal muscle weakness and arrhythmias due to cardiac muscle failure, are the main symptoms associated with excessive excursions of K^+^ levels, whether they increase or decrease [1],[2]. Hyperkalemia or hypokalemia causes symptoms such as sensory disturbances and autonomic neuropathy in the peripheral nervous system and epileptic events and disturbance of consciousness in the central nervous system [3].

Excess K^+^ ingested by an individual is promptly excreted within their urine; however, until excretion occurs, it is temporarily taken up by the cells in the body, and the K^+^ concentration in the interstitium and blood is regulated to prevent a sharp increase; this process is assisted by insulin and adrenaline [4]. This suggests that the K^+^ concentration in some cells significantly changes with each meal. It is challenging to imagine that repeating this process for several decades could damage the cells or cause aging. Our previous research showed that intracellular K^+^ concentration influences the degree of mitochondrial depolarization, which triggers cell death [5],[6]. The larger the K^+^ concentration difference between the cytosol and mitochondrial matrix, the greater the degree of mitochondrial depolarization associated with a mitochondrial permeation transition pore opening, which may induce neuronal cell death [5],[6].

Therefore, a detailed investigation of intracellular K^+^ concentration changes and further tracking of the effects of K^+^ concentration changes on intracellular organelles may be important to understand cell viability and aging. Although it is plausible that K^+^ concentrations change within the central nervous system change, for example, by gradually increasing or decreasing the expression of transporters during aging, investigations are lacking. It is possible that we are unaware of its importance; however, the reason for this may be that there is no easy way to measure K^+^ concentrations.

The whole-cell patch-clamp method can artificially keep the intracellular K^+^ concentration constant; however, it is unsuitable to measure the actual physiological concentrations of potassium. Recently, fluorescent indicators have been developed and used; however, they are unsuitable for quantifications. Classically, atomic absorption spectrophotometry is conventionally used after the cell components have been carbonized; however, this process takes time. The electrode method uses electrodes to measure the K^+^ concentration in solution; however, the scale is too large to be used at the cellular level. In recent years, electrode devices have been developed, including a device, namely the LAQUAtwin K-11, that can measure the K^+^ concentration on a small scale of approximately 300 µL; however, it is a little less sensitive. Therefore, we investigated whether we could establish a method to determine the intracellular and tissue potassium levels using this device.

## 2. Materials and methods

### 2.1. Chemicals

Each concentration of potassium chloride (KCl) was prepared by dilution from a 3.33 mM KCl standard solution (HORIBA, Ltd., Kyoto, Japan). Bovine serum albumin (BSA), glucose, lecithin, cholesterol, valinomycin, and Dulbecco's Modified Eagle Medium (DMEM) were purchased from NACALAI TESQUE Inc. (Kyoto, Japan). A 1:1:1:1 mixture of deoxynucleotide triphosphates (dNTPs) (Thermo Fisher Scientific Inc., Waltham, MA, USA) containing the same concentrations of dATP, dGTP, dCTP, and dTTP (e.g., 10 mM dNTPs contain 2.5 mM each) was applicated. Calf thymus DNA was purchased from Merck (Darmstadt, Germany). All the other materials used were standard materials.

### 2.2. Measurement of potassium concentration

An ion meter (LAQUAtwin K-11) was obtained from HORIBA Ltd. (Kyoto, Japan). The official measurement range was 4–9900 ppm and was provided in two significant figures. The measurements were performed according to the manufacturer's instructions. Each day, a calibration using 150 ppm and 2000 ppm KCl was performed. The measurements were performed by placing 300 µL of the solutions on the electrode. After measuring the biological constituents, the sensor was exposed to a neutral detergent for 5 min and thoroughly rinsed with water. Since the molecular weight of K^+^ is 39.10, 1 ppm K^+^ equals 39.10 times smaller than 1 mM K^+^, and 1 mM K^+^ equals 39.10 ppm K^+^.

### 2.3. Neuro2A cells

The cells were shared for a fee by the National Institutes of Biomedical Innovation, Health, and Nutrition (Osaka, Japan). The cells were grown in a complete medium of DMEM which contained 100 units/ml penicillin, 100 µg/ml streptomycin, and 10% fetal bovine serum at 37 °C with 5% CO_2_ and passed every 2–3 days *in vitro*. During passage, adherent cells on the culture plate were incubated with 0.05% trypsin and 1 mM EDTA. The density of the detached cells was determined, and the cells were seeded at an appropriate density for passaging.

### 2.4. Preparation of the buffer containing different concentrations of potassium ions

Hank's balanced salt solution (HBSS) contained D-glucose (5.56 mM), KCl (5.33 mM), potassium dihydrogenphosphate (0.44 mM), sodium chloride (NaCl, 138 mM), sodium hydrogen carbonate (4.17 mM), and di-sodium hydrogenphosphate (0.37 mM). Therefore, the total K^+^ concentration within the HBSS was approximately 5.8 mM. Based on HBSS, a “0 mM” buffer was prepared without KCl and potassium dihydrogenphosphate. KCl was added to the “0 mM” buffer to prepare a “30 mM” buffer.

### 2.5. Cell Incubation

The cells were detached, and 25, 50, and 100 × 10^4^ cells were aliquoted into tubes. After centrifugation, the supernatant was replaced with one of the following solutions (K^+^ [mM]): DMEM (5.3), HBSS (5.8), “0 mM” buffer, or “30 mM” buffer. The cells were gently mixed and incubated at 37 °C with 5% CO_2_ for 30 min. Valinomycin exposure was performed during the incubation. Then, the cells were centrifuged and lysed in the “0 mM” buffer by sonication. Then, the lysates were subjected to measurements.

### 2.6. Animals

The protocol used in this study followed the guidelines of the Japanese Society for Pharmacology and was approved by the Committee for the Ethical Use of Experimental Animals at Setsunan University (K22-14, K23-12). C57BL/6 female, 6-week-old mice were reared in plastic breeding cages with a light-dark cycle of 12–12 h and a humidity of 55% at 23 °C. The animals had free access to food and water. After decapitation, their brains were immediately dissected into eight regions on an ice-cold dissecting board [7]. Each tissue was weighed and subsequently lysed using sonication in the “0 mM” buffer.

### 2.7. Data analysis

The measurements were repeated four times, and the data are presented as the mean ± standard error. Student-t, Dunnett's, or Bonferroni tests were used when multiple comparative tests were required, and the figure legends notate which test was used is noted.

## 3. Results

**Figure 1.**
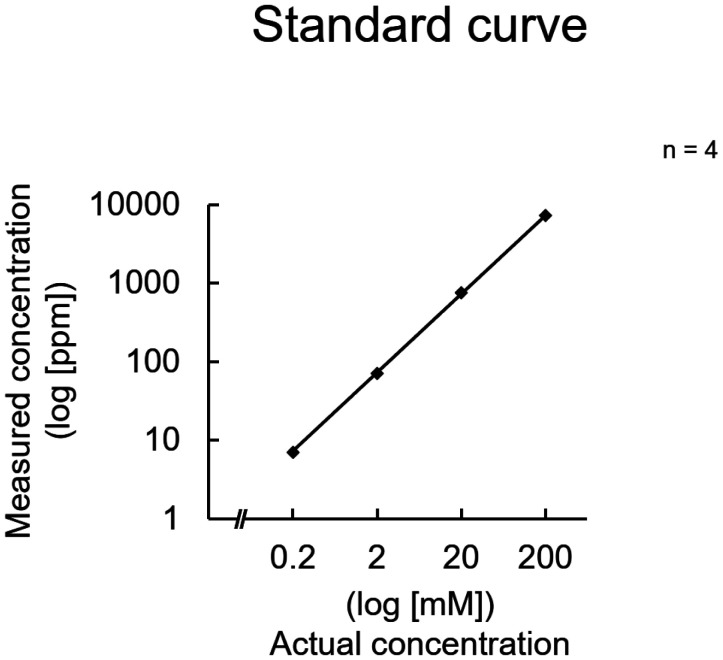
Standard curve of the quantification of potassium ions. The KCl solutions were prepared at the indicated concentrations, and their potassium ion concentrations were measured using an ion meter (LAQUAtwin K-11). The logarithm of the actual concentration of the prepared solution is plotted on the horizontal axis, and the logarithm of the measurement result is plotted on the vertical axis. Data were collected four times, and the mean value and standard error were calculated.

**Figure 2.**
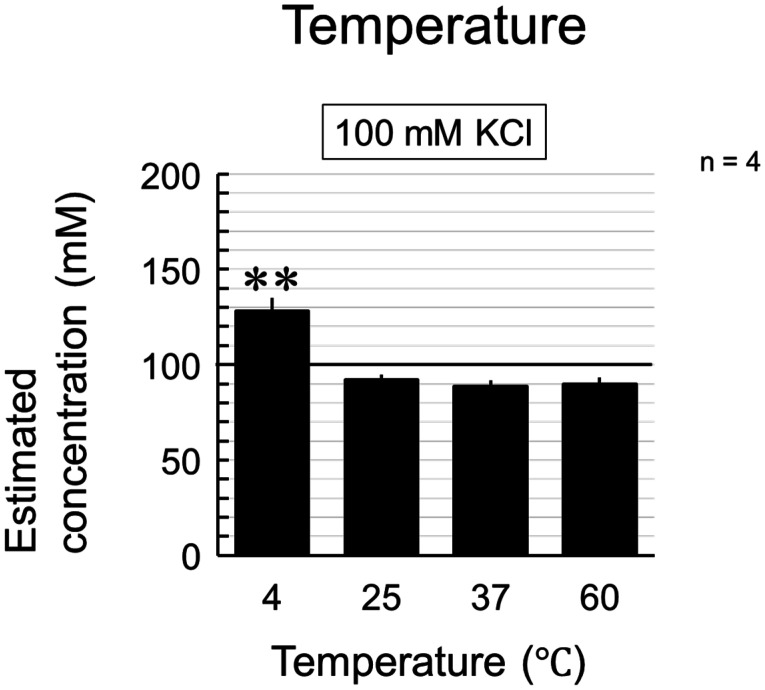
Effect of temperature on the measurement. KCl (100 mM) was used for measurements at different temperatures. The estimated concentrations (mM) were calculated from the measured concentrations (ppm). Data were collected four times, and the mean value and standard error were calculated (**P < 0.01 vs. the value at 25 °C (Dunnett's test)).

With the LAQUAtwin K-11 ion meter, the actual concentration and measured value almost matched, and the measurement was linear (Figure 1). The measurements performed on the 100 mM KCl solutions yielded a constant value at temperatures above 25 °C, although the estimated concentration was slightly below 100 mM. The measured values were significantly higher at 4 °C compared to other temperatures, which suggests that cooling is not recommended (Figure 2). Therefore, subsequent measurements were performed at 25 °C.

**Figure 3.**
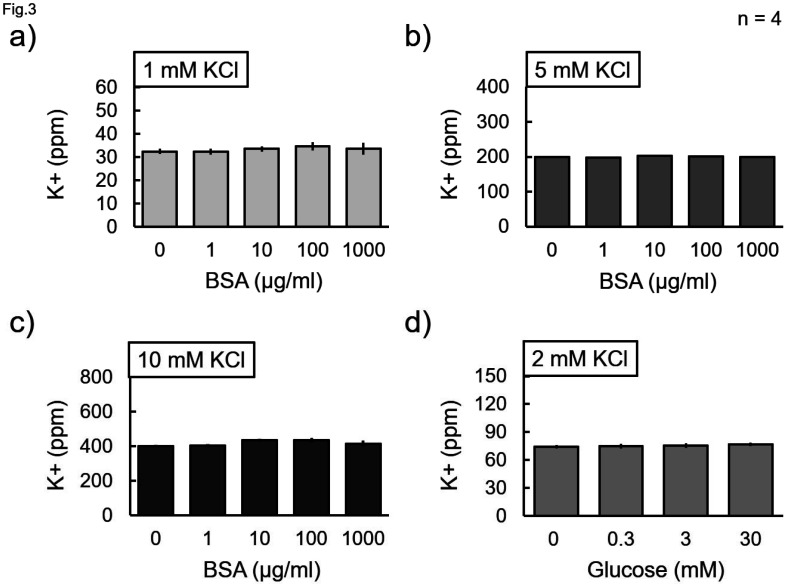
Effect of BSA and glucose on the measurement. a) 1 mM, b) 5 mM, c) 10 mM, or d) 2 mM KCl with different concentrations of a-c) bovine serum albumin (BSA) or d) glucose, as indicated, was subjected to the measurement. Data were collected four times, and the mean value and standard error were calculated. There was no significant difference between the conditions (Bonferroni test).

**Figure 4.**
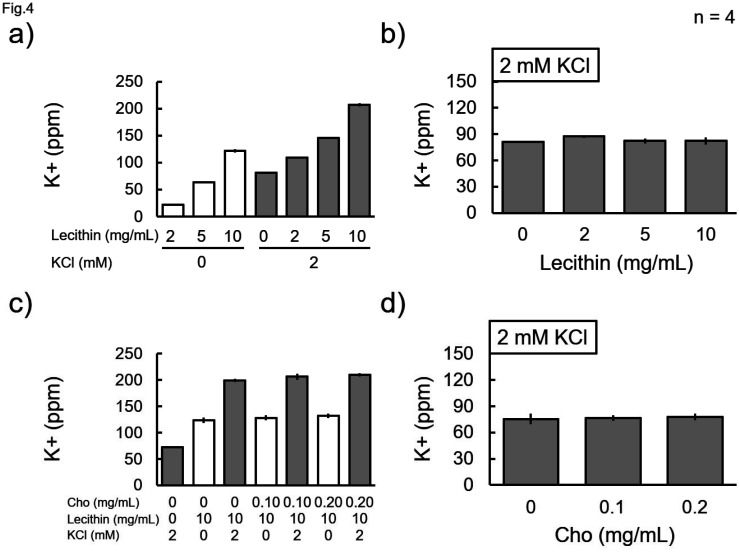
Effects of lipids on the measurement. a, c) Different concentrations of lecithin and/or cholesterol (Cho), as indicated, with or without 2 mM KCl were subjected to the measurement. The effect of b) lecithin alone, from the results of a), and the effect of d) Cho alone, from the results of b), on the measurements were calculated. Data were collected four times, and the mean value and standard error were calculated. b, d) There was no significant difference between the conditions (Bonferroni test).

**Figure 5.**
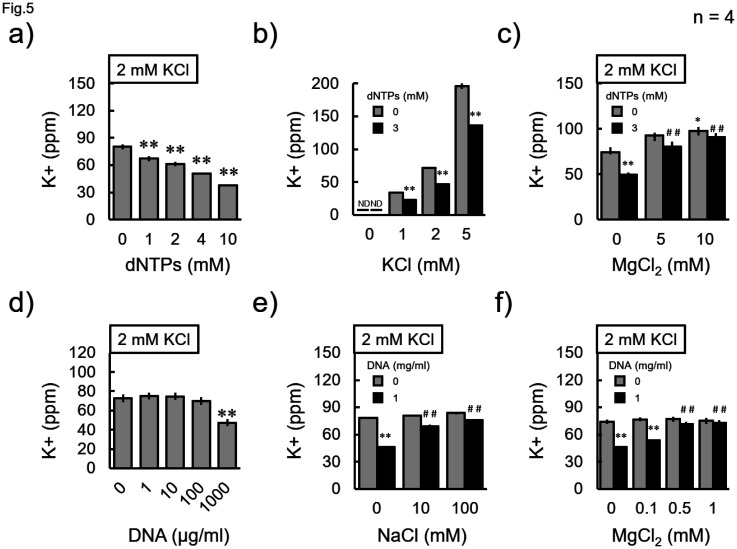
Effect of nucleotides on the measurement. a, d) Measurements were performed on solutions of 2 mM KCl with different concentrations of a) deoxynucleotide triphosphates (dNTPs) or d) calf thymus DNA, as indicated. b) Different concentrations of KCl were subjected to measurements with or without 3 mM dNTPs, as indicated. c, e, f) Effect of c, f) MgCl_2_ or e) NaCl against the c) dNTP- or e, f) DNA-dependent inhibition of the measurement. Data were collected four times, and the mean value and standard error were calculated. “ND” indicates when a value is not displayed. a, c) **P < 0.01 vs “0” (Dunnett's test), b) **P < 0.01 vs without nucleic acid at each concentration of KCl (student-t test), c, e, f) *P < 0.05, **P < 0.01 vs 2 mM KCl alone without nucleic acids or any additional salts; ^##^P < 0.01 vs 2 mM KCl with nucleic acids without any additional salts (Bonferroni test).

The effect of the presence of biological constituents on the potassium concentration measurements was investigated (Figures 3–5). The measurement with the 100 mM KCl solution was lower than the theoretical value (Figure 2); however, the measurements with the 1–10 mM KCl solutions almost agreed with the theoretical values (Figures 3–5), which suggests that the lower concentrations can be measured with higher accuracies. Although BSA was used as a representative protein, it did not change the measured value at any concentration of KCl (1, 5, and 10 mM) (Figures 3a–c). Glucose was chosen as the saccharide candidate; however, the measurements were not changed by its admixture (Figure 3d). Lecithin was chosen as the representative phospholipid because isolated lipids have a low water solubility. Lecithin contains potassium; however, when 2 mM KCl was added to it, the measured value increased by the amount added (Figure 4a). Therefore, subtraction suggested that lecithin did not affect the potassium measurements (Figure 4b). Similarly, purified cholesterol solubilized in lecithin was examined for its effect on the measurements; however, cholesterol alone did not change the measured values (Figure 4c and d).

In contrast, nucleic acids affected these measurements (Figure 5). Each concentration of dNTPs significantly reduced the measured value in a concentration-dependent manner (Figure 5a). A constant inhibition of the measured value by 3 mM dNTPs was observed at all concentrations of KCl (Figure 5b). This inhibition was suppressed by the addition of MgCl_2_ (Figure 5c). Furthermore, 1000 µg/ml calf thymus DNA, which was the highest concentration in this experiment, significantly reduced the measured value (Figure 5d). DNA inhibition was completely suppressed by the addition of either NaCl or MgCl_2_ (Figures 5e–f). In addition, it was found, by chance, that a high concentration of magnesium ions (Mg^2+^, 10 mM) enhanced the measured value (Figure 5c). MgCl_2_ itself did not cause the device to display any values (Figure 5b).

**Figure 6.**
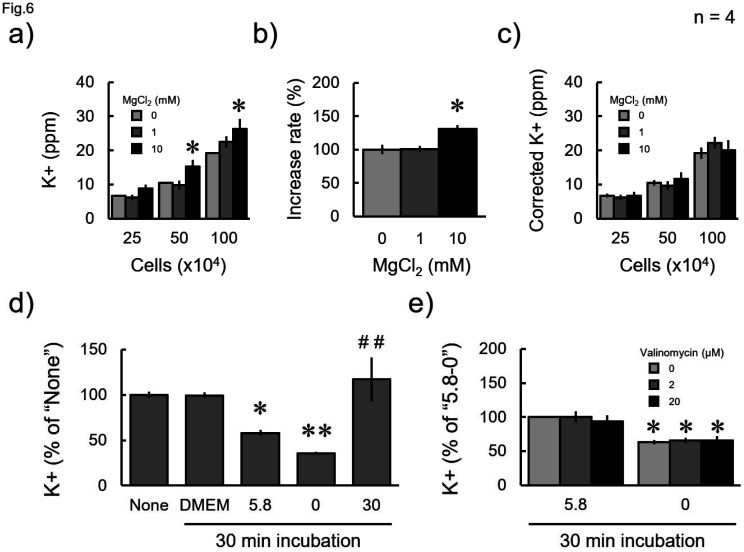
Potassium ion levels in the cell lysate as measured by the ion meter. a, c) Cells were detached from culture dishes, prepared to the indicated cell numbers, and sonicated. a) Raw or c) corrected K^+^ concentrations in the lysates, which were measured with or without MgCl_2_ at an indicated concentration. b) Increased rate of obtaining a K^+^ measurement value of 2 mM after addition of Mg^2+^. d, e) Cells were lysed after being detached from the culture dish (None) or after additional in vitro incubation for 30 min in a fresh complete medium (DMEM) or a buffer containing K^+^ at a specified concentration with different concentrations of valinomycin, as indicated. They are then lysed and applied to the ion meter. Data were collected four times, and the mean value and standard error were calculated. b) *P < 0.05 vs “0” (Dunnett's test); a, c) *P < 0.05 vs without MgCl_2_ at each cell density (Dunnett's test); d, e) *P < 0.05, **P < 0.01 vs d) “None” or e) “5.8-0,” which is a solution of 5.8 mM K^+^ and 0 mM valinomycin; d) ^##^P < 0.01 vs “5.8” (Bonferroni test).

**Figure 7.**
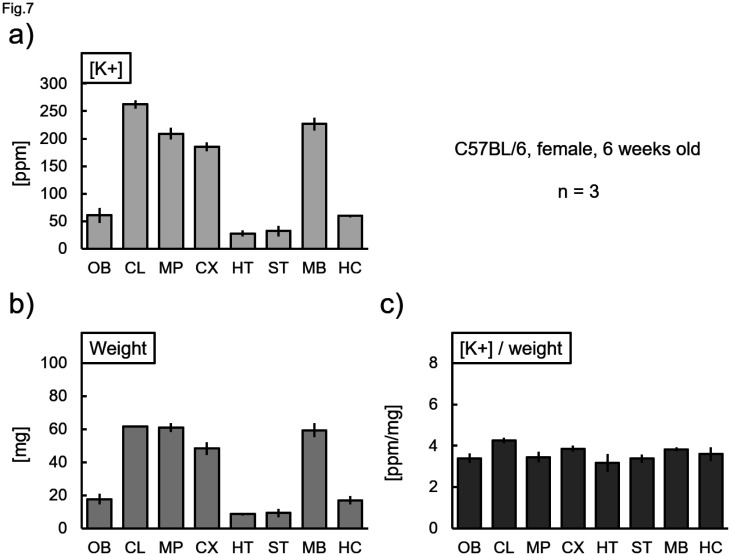
Potassium ion content in eight regions of mouse brains. Mouse brains were dissected into eight regions: the olfactory bulb (OB), the cerebellum (CL), the medulla-pons (MP), the cerebral cortex (CX), the hypothalamus (HT), the striatum (ST), the midbrain (MB), and the hippocampus (HC). Each tissue sample was weighed, lysed, and analyzed using an ion meter. a) K^+^ concentration and b) tissue weight of the regions were evaluated. c) The K^+^ concentrations per unit weight were calculated and compared. There were no significant differences between the regions (Bonferroni test).

Next, we measured the K^+^ concentrations within the cell lysates. The K^+^ concentration increased in a cell density-dependent manner. These values increased with the addition of 10 mM MgCl_2_ (Figure 6a). As shown in Figure 5c, the value increases in the presence of 10 mM MgCl_2_. Therefore, the measured value of 2 mM K^+^ alone was taken as 100%, and the % when Mg^2+^ of each concentration was added was calculated (Figure 6b). Then, the values in Figure 6a were corrected by dividing them by the values in Figure 6b (Figure 6c). Thereafter, the K^+^ value did not change under any measurement condition (Figure 6c). Next, the cells detached from the culture dish were either incubated in the complete medium (DMEM) or buffers containing different K^+^ concentrations. The measured value did not change after incubation in DMEM, though the value significantly changed in a concentration-dependent manner with the incubation in the defined salt solutions (Figure 6d). In contrast, the K^+^ concentration in the cells did not change after incubation in the presence of the potassium ionophore valinomycin (Figure 6e). Eventually, we confirmed that this method was applicable for the measurement of K^+^ concentrations in brain tissues. The potassium concentration per tissue weight was almost the same, without significant differences between the brain regions (Figure 7).

## 4. Discussion

Electrophysiological methods are the most well-known and effective methods for tracking intracellular K^+^ concentration changes [8]. However, they require expensive devices, complex and precise calculations, and highly skilled personnel. We believe that we have succeeded in establishing a method to quantify the intracellular K^+^ concentrations using a simple and inexpensive device. Volumes of 300 µL were sufficient for our method, which is a beneficial difference compared to traditional methods which utilize glass electrodes and require sample volumes in milliliters. In addition, our method is better than electrophysiological methods because it can directly measure the total K^+^ concentrations in tissue samples.

However, this method has three limitations. First, the device should not be exposed to ice-cold temperatures; because the measurement does not rely on enzymatic reactions, this is not a major problem. Second, the values are relative, and they cannot precisely define the actual intracellular K^+^ concentration. It requires information about the cytosolic volume of individual cells. This finding is potentially important. Third, although the device can be explored within a linear quantitation range, its measurements only have two significant figures. Therefore, further developments are required to enable the measurement of small volumes with a higher sensitivity.

Fluorescent indicators are being developed for intracellular K^+^ measurements and dynamic investigations associated with channel openings. We previously used a thallium ion (Tl^+^) fluorescent indicator, FluxOR™ Potassium Ion Channel Assay (Thermo Fisher Scientific Inc., Waltham, MA), to track K^+^ channel openings in living cells [6]. Although this method could measure a K^+^ channel opening, it did not track changes in the actual intracellular K^+^ concentration. Additionally, we used the K^+^ fluorescent indicator ION Potassium Green-2 AM (Abcam, Cambridge, UK) to indicate the presence of K^+^ [6], though it did not track dynamic changes or show absolute K^+^ concentrations. Therefore, we still have to await the development of highly versatile fluorescent indicators. Although the method we introduced here cannot monitor dynamic changes, it can measure static and instantaneous K^+^ concentrations in cells and tissues.

Because the final goal was to measure the K^+^ concentration in tissue homogenates of the brain, we used molecules that could replace the constituents of cells to determine any interference with the measurement. No interference from substances other than nucleic acids was observed. Although there is room to consider the suitability of the representatives, we have not observed any interference for the K^+^ concentration measurements of the cell lysates or tissue homogenates, and we stopped further searches for interfering components. The decrease in the measured K^+^ concentration in the presence of nucleic acids may simply be due to the acidic regions of the nucleic acids, that is, phosphate groups which retain K^+^. Sodium ions (Na^+^) and Mg^2+^ must have antagonized this process, which caused the measured K^+^ to return to the theoretical value. Nucleic acids have a higher affinity for Mg^2+^ than for Na^+^; therefore, Mg^2+^ at low concentrations may have suppressed the interference of nucleic acids with K^+^ measurements [9]. During the measurement of the K^+^ concentration in aqueous solutions, the presence of nucleic acids lowered the measured values. However, the amount of nucleic acid in the cell lysate was sufficiently low; therefore, it did not interfere with the measurement. The percentage of nucleic acids in human cells is only approximately 2% [10]. These results suggest that it is possible to measure the K^+^ concentration in cells and tissue lysates, while almost ignoring the measurement interference caused by nucleic acids.

Neuro2A cells were used as a representative neuronal cell line, and it was confirmed that the intracellular K^+^ concentration changed in accordance with alterations of the extracellular K^+^ concentration. Incubation with “0 mM buffer” significantly reduced the K^+^ concentration, but not to 0 ppm, which suggests that there is a substance that retains K^+^ inside the cell. As mentioned previously, nucleic acids in living cells, such as DNA and RNA, may bind K^+^. The increased standard error upon incubation with an extracellular K^+^ concentration of 30 mM (Figure 6d) may be due to the destabilization of the cell membrane because elevated extracellular K^+^ concentrations cause depolarization, even in non-excitable Neuro2A cells. In the central nervous system, astrocytes have been proposed to interfere with K^+^ in the interstitial fluid. Astrocytes temporarily retain K^+^ released by neuronal excitation, release it to neurons when appropriate, and excrete it into the blood [8]. This prevents the K^+^ released from the nervous system from drifting outside the cell for too long. This may be applicable to studies that monitor the intracellular K^+^ concentration of astrocytes.

Contrary to our expectations, exposure to valinomycin did not decrease the intracellular K^+^ concentrations (Figure 6e). Valinomycin increases the K^+^ permeability of cell membranes, induces hyperpolarization, and suppresses neurosecretion [11]. In addition, it increases the K^+^ permeability of the mitochondrial inner membrane, which causes mitochondrial depolarization under similar experimental conditions [12],[13]. Although different cell lines may have different sensitivities to valinomycin, we measured mitochondrial depolarization under similar conditions [13]. One speculative hypothesis is that the K^+^ released outside the cell is either taken up immediately or remains around the cell membrane and may not be removed by medium exchange. Currently, we are investigating why valinomycin does not decrease intracellular K^+^ concentrations. In this study, we investigated cell suspensions containing detached adherent cells, whereas we are currently investigating adherent cells. Moreover, we are exploring the conditions under which the intracellular K^+^ concentration changes using K^+^ channel openers and gate inhibitors.

Subsequently, homogenates of mouse brain regions were used. Finally, our method is obviously applicable to all types of cells and tissues. The K^+^ concentration per unit tissue weight in each brain area remained constant. Normally, the intracellular K^+^ concentration is more than 30 times higher than the extracellular space. When we obtain the changes in K^+^ concentration in tissues, it will be necessary to identify whether the detected concentration difference is of an intracellular or am extracellular origin. However, at the same time, changes in the intracellular K^+^ concentration will cause major changes. Furthermore, since the extracellular concentration correlates with the blood concentration, it is possible to confirm that the extracellular K^+^ concentration is constant by using the plasma K^+^ concentration as an extracellular reference. The liver sometimes takes up K^+^ to prevent an increase in the extracellular K^+^ concentration [4]; however, this has not been demonstrated in brain tissues. Astrocytes buffer K^+^ that enters the brain [8]. If they do not, then it can have a fatal effect on the excitatory role of neurons. For example, changes in the extracellular K^+^ concentrations have been observed in patients with renal failure and older people with reduced renal function. Although the causal relationship between renal function and cognitive decline in the older adults remains unclear, they are possibly correlated [14]. We hypothesized that intracellular K^+^ fluctuations indicate neuronal fragility [5],[6]. This measurement system can possibly be used to investigate the steady-state K^+^ levels in the central nervous system and to examine their impact on the development and progression of various neurodegenerative diseases such as dementia, amyotrophic lateral sclerosis, and Parkinson's disease, as well as psychiatric disorders such as depression and schizophrenia.
